# Receiving advice from mothers and depressive symptoms in midlife: exploring the moderating roles of gender and relationship quality

**DOI:** 10.1093/geronb/gbaf241

**Published:** 2025-12-01

**Authors:** Robert T Frase, J Jill Suitor, Megan Gilligan, Destiny Ogle, Ranran He

**Affiliations:** School of Anthropology, Political Science, and Sociology, Southern Illinois University, Carbondale, Illinois, United States; Department of Sociology, Center on Aging and the Life Course, Purdue University, West Lafayette, Indiana, United States; Department of Human Development and Family Science, University of Missouri, Columbia, Missouri, United States; Department of Sociology, Center on Aging and the Life Course, Purdue University, West Lafayette, Indiana, United States; Department of Sociology, Center on Aging and the Life Course, Purdue University, West Lafayette, Indiana, United States

**Keywords:** Social support, Intergenerational relationships, Mental health, Multilevel or hierarchical models

## Abstract

**Objectives:**

Advice has been conceptualized as both a form of support and as a threat to recipients’ feelings of autonomy and competence. However, little is known about the effects of advice from mothers on adult children’s psychological well-being. This study examines the association between the frequency of advice from mothers and adult children’s depressive symptoms. In addition, we investigate the ways in which this association is shaped by the gender of the adult child and the quality of the mother–child relationship.

**Methods:**

The sample included 687 adult children (mean age: 49.1) in 283 families collected as part of the Within-Family Differences Study. We used multilevel modeling to account for the nested structure of the data.

**Results:**

Using the full sample, we found that more frequent advice from mothers was associated with adult children’s higher depressive symptoms. Models stratified by gender revealed that advice from mothers was associated with higher depressive symptoms among sons, but not daughters. Moderation analyses found that, among the full sample, mother–adult child tension exacerbated the association between advice from mothers and adult children’s depressive symptoms. Gender comparisons revealed that, among sons, receiving advice was more strongly associated with greater depressive symptoms when tension was high or emotional closeness was low; however, neither closeness nor tension moderated the association between advice from mothers and daughters’ depressive symptoms.

**Discussion:**

This study contributes to the literature on intergenerational relations by highlighting the ways in which gender and relationship quality shape the association between receiving advice and well-being in midlife.

Parents are highly invested in the success and well-being of their offspring (Fingerman et al., 2012a), typically providing support to their children across the adult life course (Suitor et al., 2015). One form of support that parents are most likely to provide to their adult children is advice (Fingerman et al., 2012b). Advice is often conceptualized as a facet of assistance or emotional support (Bassuk et al., 2002; Ward, 2008), and in some cases, advice from parents has been found to be associated with adult children’s higher positive mood (Wang et al., 2021). However, empirical and theoretical work suggests that advice also has the capacity to undermine recipients’ feelings of autonomy and competence (Brown & Levinson, 1987; Goldsmith & Fitch, 1997; Nozaki & Gross, 2025; Smith & Goodnow, 1999), thus potentially jeopardizing well-being.

This article has two central aims. The first aim is to explore whether more frequent advice from mothers is associated with adult children’s depressive symptoms in midlife. Although receiving advice may have both positive and negative consequences, we draw from social psychological theories of “face” (Brown & Levinson, 1987; Goffman, 1955) to hypothesize that advice from mothers will be associated with higher depressive symptoms among midlife adult children. Our decision to focus on the potentially adverse association between advice and well-being is guided by existing research, which reports that negative elements of relationships are patrticularly impactful on well-being (Mavandadi et al., 2007; Newsom et al., 2003). Moreover, the association between advice from mothers and adult children’s depressive symptoms may be especially relevant in midlife. Research has shown that midlife adults tend to fare worse regarding their mental health and well-being relative to both younger and older adults (Blanchflower, 2021; Blanchflower & Graham, 2022; Carr, 2022); thus, it is crucial to understand the factors that shape depressive symptoms at this stage of the life course. Given that advice is one of the most frequently exchanged forms of support between midlife children and their parents (Huo et al., 2018) and could pose a threat to receivers’ well-being, understanding the association between advice from mothers and midlife adult children’s depressive symptoms can shed important light on the role of social relations, particularly family relations, in well-being at this point in the life course. We limit our focus to advice from mothers because they are more central in midlife adults’ social networks than are fathers (Antonucci et al., 2004), and empirical work has shown that negative interactions with more central network members have a greater impact on well-being (Cheng et al., 2011).

The second aim is to explore characteristics of adult children and their relationships with their mothers that may condition the association between advice and adult children’s depressive symptoms. Because gender plays a central role in intergenerational relations (Fingerman et al., 2020; Suitor et al., 2015), we consider the strength of the association between the frequency of advice from mothers and depressive symptoms by adult children’s gender. Further, because parent–adult child relationship quality has been found to influence adult children’s evaluations of the advice they receive from their parents (Guntzviller et al., 2017; Wang et al., 2021), we assess the roles of emotional closeness and tension in these processes. To address these questions, we use data collected from 687 adult children nested within 283 families as part of the Within-Family Differences Study (WFDS).

## Is advice really support?

Traditionally, the exchange of advice has been conceptualized as a “positive” dimension of interpersonal interaction and is often combined with items asking about whether the role partner provides reassurance and listens to the individual’s concerns to create measures of assistance or emotional support (Bassuk et al., 2002; Ward, 2008). However, scholars have questioned this conceptualization and asked whether advice may be a “negative” dimension or both a positive and negative dimension, depending upon whether the advice is wanted or unwanted (e.g., August et al., 2007; Birditt et al., 2009; Fiori et al., 2013; Wang et al., 2021). Although negative exchanges have been shown consistently to be associated with lower well-being (e.g., August et al., 2007; Fiori et al., 2013), because studies of support have often combined advice with other measures of “emotional support” or “negative exchanges,” it remains unclear whether the receipt of advice itself plays a role in psychological well-being. However, there are several bases on which to suggest that advice can have detrimental effects.

Some research has suggested that receiving support is associated with feelings of guilt and dependence (Lu & Argyle, 1992) and that high levels of received support or support characterized by the receiver as excessive are associated with lower well-being (Brock & Lawrence, 2009; Silverstein, Chen, & Heller, 1996; Williamson et al., 2019). We propose that advice, in particular, may adversely impact receivers’ well-being because of its potentially “face-threatening” qualities (Nozaki & Gross, 2025). Goffman (1955) conceptualized an individual’s “face” as the positive social value they claim for themself in interactions. When others act in ways that affirm the face claimed by an individual, that individual can be said to “have face.” However, someone’s face is *threatened* when others act or respond in ways that may undermine it. Building from Goffman’s work, Brown and Levinson’s (1987) politeness theory highlights two specific face needs; positive face, which is the desire for acceptance and approval from others (Brown & Levinson, 1987), including the desire to have one’s competence acknowledged by others (Hastings & Castle Bell, 2018), and negative face, which is the desire to be autonomous and unimpeded in one’s actions (Brown & Levinson, 1987). Feelings of competence and autonomy are both correlated with higher well-being (Reis et al., 2000). Advice can threaten the receiver’s positive face by calling their competence into question (Brooks et al., 2015; Goldsmith & Fitch, 1997). Advice can also threaten negative face, especially when it is perceived as directive, intrusive, or controlling by the recipient (Brown & Levinson, 1987; Deci & Ryan, 2012; Goldsmith & Fitch, 1997).

Autonomy and competence are particularly salient to mother–adult child relations. Past research has shown that parents play a crucial role in shaping their children’s sense of autonomy and competence in childhood, adolescence (Grolnick & Lerner, 2023; Joussement & Mageau, 2023), and young adulthood (Ratelle & Guay, 2023). Although no work has specifically explored parents’ influence on children’s sense of autonomy and competence in midlife, mothers continue to be central figures in the lives of midlife children (Antonucci et al., 2004; Suitor et al., 2015), and individuals’ self-concepts and well-being are most strongly shaped by reflected appraisals from people to whom they are close and whose opinions they trust (Gallagher et al., 2022; Rosenberg, 1973). Therefore, we predict that more frequent advice from mothers will be associated with higher depressive symptoms among adult children in midlife.

## Moderators of the association between receiving advice and depressive symptoms

Up to this point, we have focused on the potentially adverse association between the frequency of advice from mothers and adult children’s depressive symptoms without taking into consideration the gender of adult children or mother-adult child relationship quality, both of which the literature suggests may play important roles in these processes.

### Gender

The theoretical literature on gender socialization and identity can be used to hypothesize that children’s gender will shape the strength of the association between the frequency of advice from mothers and depressive symptoms. Women are socialized from childhood to form identity standards based on connectedness, expressivity, and compassion, whereas men are socialized to form identity standards defined by autonomy, agency, and independence (Carter, 2014). Consistent with these arguments, empirical work suggests that women are more likely to seek help from others (Lee, 2002) and are more receptive to advice (Feng & MacGeorge, 2006).

These gender differences in socialization regarding identity standards are especially important to consider in the context of the intergenerational transmission of advice. Adult daughters and mothers are generally closer than adult sons and mothers (Suitor et al., 2015), and closeness to advice givers is positively associated with recipients’ receptiveness to advice (Feng & MacGeorge, 2006). Conversely, advice from mothers may contradict sons’ internalized identity standards, and people feel distress when they receive input from others that does not align with their identity standards (Stets, 2018). Therefore, advice may be especially distressing to sons. Thus, we hypothesize that the association between the frequency of advice from mothers and depressive symptoms will be stronger among sons than daughters.

### Closeness and tension

Theoretical and empirical scholarship on relationship quality and support can be used to propose that emotional closeness and tension will shape the association between the frequency of advice from mothers and adult children’s depressive symptoms. According to Relational Regulation Theory, the relationship between the support provider and receiver shapes the receiver’s appraisals of support (Lakey & Orehek, 2011). Consistent with this argument, empirical research has shown that advice receivers’ evaluations of advice are shaped by the quality of their relationship with the advice giver. People are more receptive to advice from those to whom they are relationally close (Feng & MacGeorge, 2006). Conversely, advice is more likely to be perceived unfavorably when recipients report having more negative relationships with givers (Wang et al., 2021).

Because relationship quality is crucial to receivers’ evaluations of advice, we suggest that mother–adult child relationship quality will condition the strength of the association between the frequency of advice from mothers and adult children’s depressive symptoms. Thoits (2011) suggests that social support from people to whom the receiver is emotionally close can be especially effective at buffering the effects of stressors on physical and mental health. Thus, we predict that emotional closeness will mute the association between the frequency of advice from mothers and adult children’s depressive symptoms. On the other hand, stressors experienced simultaneously may be especially detrimental to individuals’ well-being because accumulated strains can hinder their abilities to cope (August et al., 2007; Ingersoll-Dayton et al., 1997). We posit that the stressors associated with interpersonal tension may intensify the psychological toll of receiving advice. Therefore, we predict that tension will exacerbate the association between the frequency of advice from mothers and adult children’s depressive symptoms.

In summary, we propose that (a) the association between the frequency of advice from mothers and adult children’s depressive symptoms will be weaker among children who report higher levels of emotional closeness to their mothers, and (b) the association between the frequency of advice from mothers and adult children’s depressive symptoms will be stronger among children who report higher levels of tension with their mothers.

Gender plays a central role in the quality of intergenerational relations (Fingerman et al., 2020; Suitor et al., 2015). Therefore, we also consider the moderating effects of emotional closeness and tension separately for daughters and sons. Empirical research suggests that women are more involved in, and more intensely affected by, their social relationships than are men (Birditt et al., 2009; Polenick et al., 2018; Suitor et al., 2020). Further, this pattern is especially strong regarding the impact of mothers’ relationships with daughters, relative to sons (Suitor et al., 2020). Taken together, these findings suggest that relationship quality would be more impactful on the association between advice from mothers and *daughters’* depressive symptoms. Therefore, we predict that emotional closeness and tension will significantly moderate the association between advice from mothers and depressive symptoms for daughters, but not for sons.

To summarize, we make the following predictions based on a combination of theoretical arguments and empirical findings:

H1: Receiving more advice from mothers will be associated with higher depressive symptoms among midlife adult children.

H2: The association between the frequency of advice from mothers and midlife adult children’s depressive symptoms will be stronger among sons than daughters.

H3: The association between the frequency of advice from mothers and midlife adult children’s depressive symptoms will be weaker among children who report higher levels of emotional closeness to their mothers.

H4: The association between the frequency of advice from mothers and midlife adult children’s depressive symptoms will be stronger among children who report higher levels of tension with their mothers.

H5: Closeness and tension will moderate the association between the frequency of advice from mothers and depressive symptoms for daughters, but not for sons.

## Methods

### Procedures

The data used in the present analyses were collected as part of the second wave of the WFDS. The design of the study involved selecting a sample of mothers aged 65–75 years with at least two living adult children and collecting data from mothers regarding each of their children. Massachusetts city and town lists were used as the source of the original study sample. With the assistance of the Center for Survey Research at the University of Massachusetts, Boston, we drew a probability sample of women aged 65–75 years with two or more children from the greater Boston area. The Time 1 sample consisted of 566 mothers, which represented 61% of those who were eligible for participation, a rate comparable to that of similar surveys in the 2000s (Wright & Marsden, 2010). Further details of the design can be found in Suitor et al. (2013, 2017) and at http://web.ics.purdue.edu/∼jsuitor/within-family-differences-study/, where portions of this section have already been published.

For the second wave of the study, the survey team attempted to contact each mother who participated in the original study to schedule a 60- to 90-min in-person interview. At T2, 420 mothers were interviewed. Of the 146 mothers who participated at only T1, 78 had died between waves, 19 were too ill to be interviewed, 33 refused, and 16 could not be reached. Thus, the 420 represent 86% of mothers who were living at T2. Comparisons between the mothers alive at T2 who did and did not participate revealed that those who participated were better educated and in better health. Comparing the T1 and T2 samples revealed that mothers who were not interviewed at T2 were less healthy, less educated, less likely to have been married at T1, and more likely to be Black.

Following the interview, mothers were asked for their adult children’s contact information; at T2, 81% of the mothers provided contact information—a rate higher than typically found in studies of multiple generations (Kalmijn & Liefbroer, 2011). Seventy-five percent of the adult children for whom contact information was available agreed to participate, resulting in a final sample of 826 children nested within 360 families. Semi-structured interviews with the adult children were conducted on the telephone and lasted approximately 45–60 min. Analyses comparing mothers with and without participating children revealed no differences between these two groups in terms of race, marital status, education, age, or number of children; daughters, married offspring, and those with higher education were slightly more likely to participate, consistent with other studies of multiple generations (Kalmijn & Liefbroer, 2011).

The analytic sample for this article includes 687 adult children nested within 283 families in which mothers were alive at the time of the children’s T2 interviews. Adult children in the present sample had no missing data on any analytic variables. Table 1 presents the descriptive statistics.

**Table 1.** Demographic information on families and adult children (687 adult children in 283 families).

Characteristics	Full sample	Daughters	Sons
	*N *= 687	*n *= 392	*n *= 295
**Family-level characteristics**			
** Mother’s age (mean, *SD*)**	77.83 (3.11)	77.91 (3.11)	77.72 (3.12)
** Mother’s education^a^ (mean, *SD*)**	4.09 (1.74)	4.04 (1.69)	4.16 (1.80)
** Nonwhite (%)**	22.27	23.72	20.34
**Child-level characteristics**			
** Depressive symptoms (mean, *SD*)**	11.55 (4.60)	11.77 (4.68)	11.25 (4.48)
** Frequency of advice from mother (mean, *SD*)**	1.36 (1.61)	1.50 (1.66)	1.18 (1.53)
** Daughter (%)**	57.06		
** Age (mean, *SD*)**	49.08 (5.72)	49.04 (5.80)	49.14 (5.62)
** Employed (%)**	80.35	77.55	84.07
** Self-rated physical health^b^ (mean, *SD*)**	3.79 (1.08)	3.76 (1.15)	3.83 (0.99)
** Married (%)**	71.76	70.15	73.90
** Education^a^ (mean, *SD*)**	5.21 (1.58)	5.15 (1.59)	5.28 (1.56)
** Closeness to mother^c^ (mean, *SD*)**	9.51 (2.18)	9.64 (2.28)	9.34 (2.04)
** Tension with mother^d^ (mean, *SD*)**	6.60 (2.30)	6.76 (2.44)	6.37 (2.07)
** Mother’s health problems (%)**	64.92	65.31	64.41

a Education variables measured as 1 = eighth grade or less, 2 = 1–3 years of high school, 3 = high school graduate, 4 = vocational or other non-college post-secondary education, 5 = 1–3 years of college, 6 = college graduate, 7 = graduate work.

b Self-rated health measured as 5 = excellent, 4 = very good, 3 = good, 2 = fair, 1 = poor.

c Closeness to mother measured on a scale ranging from 3 to 12, with higher scores denoting greater closeness.

d Tension with mother measured on a scale ranging from 3 to 12, with higher scores denoting greater tension.

### Measures

#### Dependent variable

To measure adult children’s *depressive symptoms*, we used a 7-item version of the Center for Epidemiological Studies Depression (CES-D) scale (Ross & Mirowsky, 1984), which asks, “Next is a list of sentences that describe how you may have felt or behaved in the past week. After each sentence please tell me how many days since last (DAY OF WEEK) you felt this way: (a) you felt you couldn’t get going; (b) you felt sad; (c) you had trouble getting to sleep or staying asleep; (d) you felt that everything was an effort; (e) you felt lonely; (f) you felt you couldn’t shake off the blues; and (g) you had trouble keeping your mind on what you were doing.” Each item was measured on a 4-point scale ranging from “less than 1 day” (1) to “5–7 days” (4). We summed these items to create a scale that ranged from 7 to 28, with higher scores denoting higher depressive symptoms (mean = 11.55, *SD* = 4.60, Cronbach’s alpha = 0.84).

#### Independent variable

*Advice from mother* was measured by first asking respondents, “In the past year, has your mother given you advice on a decision you had to make?” Respondents who answered “yes” were then asked, “About how often did she help you–once or twice, 3–5 times, 6–10 times, 11–20 times, or more than 20 times?” We utilized respondents’ answers to these questions to create a scale ranging from “never received advice from mother” (0) to “received advice from mother more than 20 times” (5).

#### Moderators

Adult children’s *gender* was coded as 1 = daughter, 0 = son.

We created a measure for *emotional closeness* by combining the following three items: (a) Using any number from 1 to 7, where 1 is very distant and 7 is very close, what number would you use to describe the relationship between you and your mother nowadays? (b) How often does your mother make you feel loved or cared for?—very often (5), fairly often (4), sometimes (3), rarely (2), or never (1)? (c) Being with your mother makes you feel very happy–strongly agree (4), agree (3), disagree (2), strongly disagree (1)? We collapsed the lowest categories of the first two items, so that the scores for all three items ranged from 1 to 4. These items were summed, and the range of the combined closeness scale was 3–12, with higher scores denoting greater closeness. Three cases had missing data on one of the items. For each of these cases, we replaced this missing value with the average of the other two items. In this sample, the mean score for closeness was 9.51 (*SD* = 2.18, Cronbach’s alpha = 0.75, skewness = −0.70).

We used a similar approach to measure *tension*. The following three items were combined to create a tension scale: (a) “Sometimes no matter how close we may be to someone, the relationship can also at times be tense and strained. Using any number from 1 to 7, where 1 is not at all tense and strained and 7 is very tense and strained, what number would you use to describe how tense and strained the relationship between you and your mother is nowadays?” (b) “How often would you say the two of you typically have disagreements or conflicts—very often (5), fairly often (4), sometimes (3), rarely (2), or never (1)?” (c) “Does your mother make too many demands on you very often (5), fairly often (4), sometimes (3), rarely (2), or never (1)?” We collapsed the highest categories of all three items, so that the scores ranged from 1 to 4 to be consistent with the measure of closeness. These items were summed, and the range of the combined tension scale was 3–12, with higher scores denoting greater tension. In this sample, the mean score for tension was 6.60 (*SD* = 2.30, Cronbach’s alpha = 0.70, skewness = 0.50).

#### Covariates

##### Family-level characteristics

Mothers’ age at T2 was calculated using each mother’s own reports at T1 of the year in which they were born. *Race* was measured by asking mothers at T1 to select with which racial or ethnic group(s) they identified (e.g., White, Black or African American, Hispanic or Latina, Native American, Asian). For the present analysis, families were coded as 1 = Nonwhite, 0 = White. Mothers’ *educational attainment* was self-reported at T1; categories ranged from eighth grade or less (1) to postgraduate work (7).

##### Adult child characteristics

*Employment* was measured by asking each respondent whether he or she was currently working a job for pay (0 = no; 1 = yes). *Marital status* was coded as 0 = not married; 1 = married. Respondents’ *educational attainment* was reported by their mothers at T1; categories ranged from eighth grade or less (1) to post-graduate work (7). *Self-rated physical health* was measured on a scale ranging from poor (1) to excellent (5). To measure *children’s perceptions of their mothers’ health*, each respondent was asked whether their mother: (a) Currently needed assistance with any of several Instrumental Activities of Daily Living (IADL) or Activities of Daily Living (ADL) (i.e., light housework, transportation, food shopping, dressing, eating, bathing, and toileting); or (b) had needed assistance in the previous 2 years for a serious illness or injury (1 = mother had ADL/IADL limitations and/or a serious illness/injury for which she needed help within the last two years, 0 = mother did not).

### Analytic strategy

Because the 687 adult children were nested within 283 families, we used multilevel linear regression modeling, which accounts for nonindependence and allows for correlated error structure. The MIXED command in Stata 19 provides mixed-effect models that can include both predictors at the adult–child level and the family level (Allison, 2009).

We present a total of nine multilevel models in this article. The first model regressed adult children’s depressive symptoms on the frequency of advice from mothers and the covariates. To examine how adult children’s gender shaped the association between advice from mothers and depressive symptoms, we present separate models for daughters and sons. We compared the effects across these models based on Clogg’s test (Paternoster et al., 1998). We took this stratified approach because results using this analytic strategy are easier to present and interpret, and providing separate models shows how the set of covariates predicted depressive symptoms within daughters and sons. We introduced interaction terms between (1) the frequency of advice from mothers and emotional closeness and (2) the frequency of advice from mothers and tension to examine whether the association between advice from mothers and adult children’s depressive symptoms was moderated by either dimension of relationship quality. We again used stratified models to examine whether these interaction effects differ between sons and daughters.

## Results

Our first hypothesis predicted that the frequency of advice from mothers would be associated with higher depressive symptoms among midlife adult children. As shown in Model 1 of Table 2, this hypothesis was supported (*B* = 0.27, *p *< 0.01).

**Table 2.** Mixed effects linear regression model results predicting adult children’s depressive symptoms (687 adult children in 283 families).

Variables	Model 1: Full sample		Model 2: Daughters		Model 2: Sons	
	*N *= 687		*n *= 392		*n *= 295	
	Estimate	*SE*	Estimate	*SE*	Estimate	*SE*
**Family-level characteristics**						
** Mother’s age**	0.15**	0.05	0.14*	0.07	0.16^+^	0.08
** Mother’s education**	0.32**	0.10	0.19	0.14	0.52**	0.16
** Nonwhite**	−0.84*	0.40	−0.25	0.52	−1.64*	0.66
**Child-level characteristics**						
** Frequency of advice from mother**	0.27**	0.10	0.18 ^a^	0.13	0.47**^a^	0.16
** Daughter**	0.08	0.31				
** Age**	−0.04	0.03	−0.04	0.04	−0.06	0.05
** Employed**	−1.70***	0.42	−1.09*	0.52	−2.63***	0.69
** Self-rated physical health**	−1.35***	0.16	−1.52***	0.20	−1.10***	0.24
** Married**	−1.30***	0.35	−0.97*	0.45	−1.61**	0.55
** Education**	−0.23*	0.12	−0.33*	0.15	−0.16	0.18
** Closeness to mother**	−0.05	0.08	0.07	0.10	−0.24^+^	0.13
** Tension with mother**	0.28***	0.08	0.38***	0.09	0.10	0.13
** Mother’s health problems**	0.21	0.33	−0.17	0.43	0.71	0.49
**Model statistics**						
** Overall *R* ^2^**	0.26		0.31		0.25	
** Akaike information criterion**	3,868.21		2,206.36		1,662.92	
** Bayesian information criterion**	3,940.73		2,265.93		1,718.23	

*Note.* SE = standard error.

a The difference between the coefficients is significant at the *p *< 0.10 level.

+ *p *< 0.10.

* *p *< 0.05.

** *p *< 0.01.

*** *p *< 0.001.

We also found support for our second hypothesis. As shown in Models 2 and 3 of Table 2, the frequency of advice from mothers was associated with higher depressive symptoms among sons (*B* = 0.47, *p *< 0.01), but not daughters (*B* = 0.18, n.s.). The difference between these coefficients trended toward significance (*t* = 1.41, *p *< 0.10).

**Figure 1.**
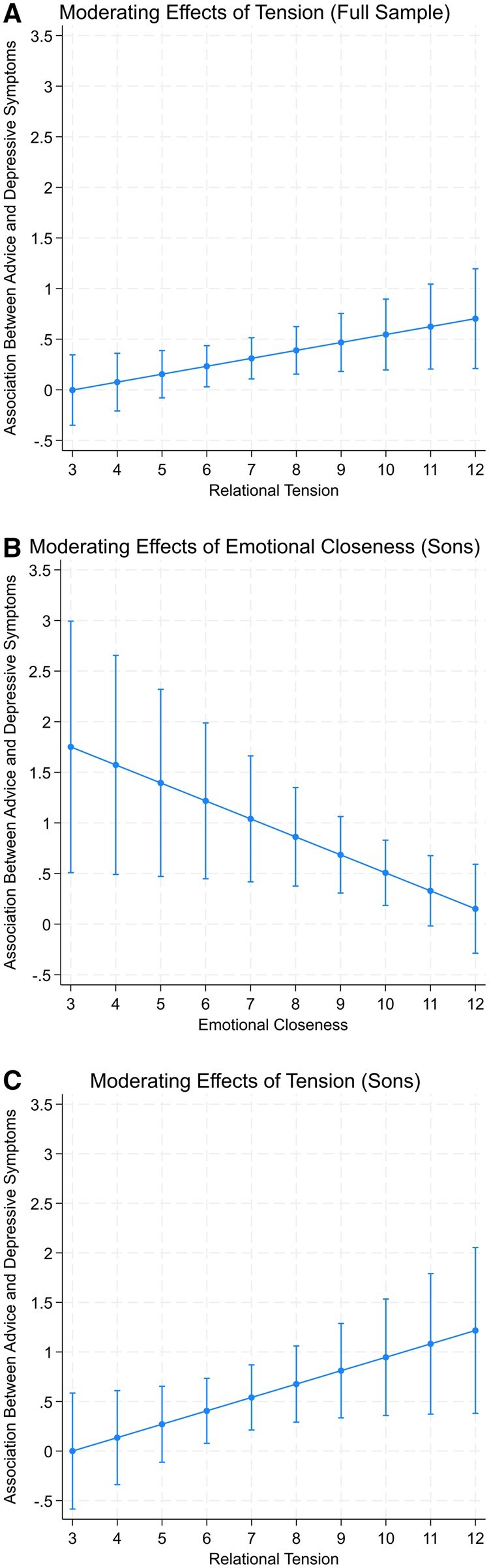
Average marginal effects of advice on depressive symptoms across the ranges of tension (A: Full Sample; C: Sons) and closeness (B: Sons).

To test our third and fourth hypotheses, we examined the interactions between (a) the frequency of advice and emotional closeness and (b) the frequency of advice and tension, using the full sample. As shown in Model 1 of Table 3, we found no moderation effects of emotional closeness (*B* = −0.05, n.s.). However, as shown in Model 2, the interaction between the frequency of advice from mothers and mother–child tension trended toward significance (*B* = 0.08, *p *< 0.10). To better understand this interaction, we plotted the average marginal effect (AME) of advice from mothers on adult children’s depressive symptoms across the full range of tension (displayed in Figure 1A). The positive slope of AMEs in Figure 1A indicates that the strength of the association between advice from mothers and adult children’s depressive symptoms increased at higher levels of tension. At high tension, we found that the association between advice and adult children’s depressive symptoms was relatively strong (Tension = 10: AME = 0.54, *p *< 0.01). The association persisted at average tension, though at roughly half the strength (Tension = 7: AME = 0.31, *p *< 0.01). However, the association between the frequency of advice from mothers and adult children’s depressive symptoms was negligible at the lowest tension (Tension = 3: AME = −0.002, n.s.). Thus, our findings suggest that tension exacerbated the strength of the association between advice from mothers and midlife children’s depressive symptoms.

**Table 3.** Mixed effects linear regression model results predicting adult children’s depressive symptoms with interactions (687 adult children in 283 families).

Variable	Full Sample *(N *= 687)				Daughters (*n *= 392)				Sons (*n *= 295)			
	Model 1		Model 2		Model 3		Model 4		Model 5		Model 6	
	*B*	*SE*	*B*	*SE*	*B*	*SE*	*B*	*SE*	*B*	*SE*	*B*	*SE*
**Family-level characteristics**												
** Mother’s age**	0.15**	0.05	0.15**	0.05	0.14^+^	0.07	0.14^+^	0.07	0.15^+^	0.09	0.17*	0.09
** Mother’s education**	0.32**	0.10	0.31**	0.10	0.19	0.14	0.18	0.14	0.52**	0.16	0.52**	0.16
** Nonwhite**	−0.82*	0.40	−0.84*	0.40	−0.25	0.52	−0.26	0.51	−1.62*	0.66	−1.69*	0.66
**Child-level characteristics**												
** Frequency of advice from mother**	0.75	0.50	−0.24	0.29	0.23	0.60	−0.13	0.35	2.28*	0.88	−0.41	0.49
** Daughter**	0.09	0.31	0.11	0.31								
** Age**	−0.04	0.03	−0.05	0.03	−0.04	0.04	−0.04	0.04	−0.06	0.05	−0.07	0.05
** Employed**	−1.70***	0.42	−1.66***	0.42	−1.09*	0.52	−1.06*	0.52	−2.47***	0.68	−2.61***	0.68
** Self-rated physical health**	−1.36***	0.16	−1.34***	0.16	−1.52***	0.20	−1.51***	0.20	−1.12***	0.24	−1.12***	0.24
** Married**	−1.28***	0.35	−1.27***	0.35	−0.97*	0.45	−0.95*	0.45	−1.58**	0.55	−1.56**	0.55
** Education**	−0.23*	0.12	−0.23^+^	0.12	−0.33*	0.15	−0.33*	0.15	−0.19	0.18	−0.16	0.18
** Closeness to mother**	−0.01	0.10	−0.06	0.08	0.08	0.12	0.06	0.11	−0.10	0.15	−0.23^+^	0.13
** Tension with mother**	0.28***	0.08	0.17^+^	0.10	0.38***	0.09	0.31*	0.12	0.06	0.13	−0.06	0.16
** Mother’s health problems**	0.19	0.33	0.22	0.32	−0.18	0.43	−0.14	0.43	0.63	0.49	0.68	0.49
**Frequency of advice from mother**												
** x Closeness to mother**	−0.05	0.05			−0.005	0.06			−0.18*	0.09		
** x Tension with mother**			0.08^+^	0.04			0.05	0.05			0.14^+^	0.07
**Model statistics**												
** Overall *R* ^2^**	0.26		0.27		0.31		0.31		0.26		0.26	
** Akaike information criterion**	3,869.27		3,866.70		2,208.36		2,207.47		1,660.72		1,661.47	
** Bayesian information criterion**	3,946.32		3,943.75		2,271.90		2,271.01		1,719.71		1,720.46	

*Note. SE* = standard error.

+ *p* < 0.10.

* *p* < 0.05.

** *p* < 0.01.

*** *p* < 0.001.

Finally, Hypothesis 5 predicted that emotional closeness and tension would moderate the association between the frequency of advice from mothers and depressive symptoms among daughters, but not sons. As shown in Models 3 and 4 on Table 3, contrary to our expectations, we found no moderation effects of emotional closeness or tension for the association between the frequency of advice from mothers and daughters’ depressive symptoms (*B* = −0.005, n.s. for closeness; *B* = 0.05, n.s. for tension). However, as shown in Models 5 and 6, we found that emotional closeness moderated the association between the frequency of advice from mothers and sons’ depressive symptoms (*B* = −0.18, *p *< 0.05), and the interaction between the frequency of advice from mothers and mother–son tension trended toward significance (*B* = 0.14, *p *< 0.10). Taken together, these findings suggest that relationship quality moderated the association between the frequency of advice from mothers and depressive symptoms among sons, but not daughters.

To better understand how closeness influenced the association between advice from mothers and sons’ depressive symptoms, we plotted the AME of advice from mothers on sons’ depressive symptoms across the full range of emotional closeness in Figure 1B. The negative slope of AMEs in Figure 1B indicates that the strength of the association between advice from mothers and sons’ depressive symptoms decreased at higher levels of closeness. At low and average levels of closeness, the association between advice from mothers and sons’ depressive symptoms was relatively large (Closeness = 5: AME = 1.40, *p *< 0.01; Closeness = 8: AME = 0.86, *p *< 0.01). However, the relationship between the frequency of advice from mothers and sons’ depressive symptoms was negligible at the highest emotional closeness (Closeness = 12: AME = 0.15, n.s.). Then, to better understand how tension influenced the association between advice from mothers and sons’ depressive symptoms, we plotted the AME of advice from mothers on sons’ depressive symptoms across the full range of tension in Figure 1C. The positive slope of AMEs in Figure 1C indicates that the strength of the association between advice from mothers and sons’ depressive symptoms increased at higher levels of tension. At high and average levels of tension, the association between advice from mothers and sons’ depressive symptoms was relatively large (Tension = 10: AME = 0.95, *p *< 0.01; Tension = 7: AME = 0.54, *p *< 0.01). However, the association between the frequency of advice from mothers and sons’ depressive symptoms was negligible at low tension (Tension = 3, AME = 0.0002, n.s.). Thus, our findings suggest that emotional closeness buffered the strength of the association between advice from mothers and midlife sons’ depressive symptoms, whereas tension exacerbated its strength.

### Sensitivity analyses

We conducted two sensitivity analyses to test the robustness of our findings. First, to assess whether only high levels of advice were associated with higher depressive symptoms, we recoded advice frequency from mothers as a categorical variable such that respondents were categorized as receiving *no advice in the last year* (referent category*), low levels of advice* (1–5 times in the last year), or *high levels of advice* (6+ times in the last year). In our analysis using this categorical measure of advice, we found that respondents who received no advice and low levels of advice had similar levels of depressive symptoms. However, compared to respondents who received no advice, respondents who received high levels of advice had higher levels of depressive symptoms. This sensitivity analysis produced the same pattern of results regarding differences in the association between advice and depressive symptoms by gender and relationship quality as the main analysis, albeit that only respondents who received high levels of advice had higher depressive symptoms than respondents who received no advice (tables not shown). This sensitivity analysis suggests that only frequent advice is detrimental to well-being.

Second, due to the low numbers of respondents who reported high levels of tension or low levels of closeness, we re-ran the models with further collapsed versions of the relationship quality scales such that the lowest three values of closeness (3–5) and the highest three values of tension (10–12) were consolidated. These models produced the same pattern of results as the main analysis (tables not shown).

## Discussion

Advice has traditionally been conceptualized as a positive dimension of social support; however, advice also has the potential to threaten face and decrease well-being. Because advice is often aggregated into broader measures of social support or negative interactions (August et al., 2007; Ward, 2008), little is known about how advice itself is associated with the well-being of recipients, especially in the context of parent–adult child relationships. The goal of this article was to extend research on intergenerational exchanges of support and well-being by focusing on advice from mothers as a potential source of psychological distress. In addition, we investigated the potential moderating roles of adult children’s gender and relationship quality in these processes.

First, drawing on theories of face (Brown & Levinson, 1987; Goffman, 1955), we predicted that the frequency of advice from mothers would be associated with adult children’s higher depressive symptoms. In the main effects analysis, we found that the frequency of advice from mothers was associated with adult children’s higher depressive symptoms, providing support for our first hypothesis. Next, wedrew on empirical work and theories of gender socialization, identity, and relational regulation (Carter, 2014; Lakey & Orehek, 2011; Stets, 2018) to predict that gender and relationship quality would shape the strength of the association between the frequency of advice from mothers and adult children’s depressive symptoms. We found support for our second hypothesis, which predicted that the association between the frequency of advice from mothers and depressive symptoms would be stronger for sons than daughters. Specifically, we found that advice from mothers was associated with higher depressive symptoms among sons, but not among daughters. Our third and fourth hypotheses proposed that the association between receiving advice and adult children’s depressive symptoms would be conditioned by relationship quality. Among the full sample, we found that the association between the frequency of advice from mothers and adult children’s depressive symptoms was exacerbated by mother–child tension; however, we did not find evidence that emotional closeness moderated this association. Finally, our fifth hypothesis predicted that relationship quality would moderate the association between the frequency of advice from mothers and depressive symptoms among daughters, but not sons. Contrary to our expectations, we found no evidence that either dimension of relationship quality shaped the association between the frequency of advice from mothers and adult daughters’ depressive symptoms. Instead, we found that both dimensions of relationship quality shaped the association between the frequency of advice from mothers and adult sons’ depressive symptoms. Specifically, emotional closeness muted the adverse association between the frequency of advice from mothers and adult sons’ depressive symptoms, whereas tension amplified this association.

There are several explanations that may account for these unexpected findings. First, as we discussed above, men are socialized to form identity standards defined by autonomy, agency, and independence (Carter, 2014) and, therefore, are less receptive to advice and less likely to seek help (Feng & MacGeorge, 2006; Lee, 2002). This resistance to support may be especially salient in the context of conflictual relationships, leading tension to play an especially potent role in exacerbating the association between advice from mothers and sons’ depressive symptoms. Similarly, men’s greater resistance may mean that emotional closeness serves an especially crucial role in neutralizing the adverse effects of advice among sons. Second, a previous study found that marital and romantic strain amplified the effects of severe impairment on negative emotions among men but not women (Carr et al., 2017). To explain these findings, Carr and colleagues (2017) suggested that because men are less likely to perceive and respond to marital difficulties, those men who do perceive and acknowledge marital strain may be especially sensitive to their marriages’ relationship quality. This explanation may also apply to the present findings. Mothers and daughters tend to have more intense relationships than mothers and sons, involving more closeness and conflict (Fingerman et al., 2020; Suitor et al., 2015). Thus, sons who perceive particularly high levels of either closeness or tension in their relationships with their mothers may be especially sensitive to the effects of those dimensions of relationship quality.

### Future directions and limitations

We hope that future research will build on this study in several directions. First, existing research has suggested that individuals’ well-being may be uniquely affected by advice that is unwanted or imposed (Van Swol et al., 2019; Wang et al., 2021). Moreover, as we discussed earlier, recipients’ evaluation of advice is shaped by the quality of the relationship with the person offering the advice (Feng & MacGeorge, 2006; Wang et al., 2021), and unwanted and unsolicited advice can be a source of tension in intergenerational relationships (Fingerman, 1996). For these reasons, we expect that unwanted and unsolicited advice would be correlated with lower closeness and higher tension between advice givers and recipients. However, the WFDS did not ask respondents whether they asked for advice from their mothers or whether they wanted the advice they received. Future research should explore the varying ways in which relationship quality conditions the effects of wanted, unwanted, solicited, and unsolicited advice on sons’ and daughters’ well-being.

Second, future scholarship in this area should consider how parents’ gender shapes the impact of advice on adult children’s well-being. Existing scholarship has documented that the gender of both parents and adult children impacts intergenerational relationship quality and support exchanges (Fingerman et al., 2020; Huo et al., 2019). Moreover, some evidence suggests that fathers and mothers provide different types of advice to children (Carlson, 2014). Given these differences, examining the unique roles that advice from mothers and advice from fathers have in shaping adult children’s well-being is a promising area for future research. However, the WFDS did not ask respondents to report on advice they received from fathers at T2.

Third, we encourage future researchers to consider variations in the association between advice from mothers and adult children’s depressive symptoms by the ages of both adult children and their parents. Carstensen’s Socioemotional Selectivity Theory posits that as individuals’ time horizons decrease, they tend to prioritize socioemotional dimensions of their lives and increasingly navigate social interactions to ensure their high emotional quality (Carstensen et al., 1999). Aging may therefore lead both parents and their adult children to more carefully navigate potentially fraught interactions, such as exchanges of advice. Thus, the negative association between advice from mothers and midlife children’s well-being may decrease with age. However, the original sample of the WFDS was designed to be comprised of mothers born within the same cohort who were in the first decade of later life (65–75). Moreover, of the 687 adult children in the analytic sample, only 38 (5.5%) fall outside of the midlife age range of 40–64. Therefore, the sample does not have the age diversity in either generation needed to test these possibilities.

Finally, we are unable to rule out the possibility of reverse causality because the data we used for this analysis were cross-sectional. For example, previous research has found that parents provide more support to adult children who experienced problems (Gilligan et al., 2017; Huo et al., 2019; Suitor et al., 2006); therefore, adult children with higher levels of psychological distress may receive more support from their mothers, including advice. Because we utilize measures of advice receipt and depressive symptoms measured at one time point, we do not interpret our findings as causal estimates due to potential issues of temporal ordering.

### Conclusion

In sum, the findings presented in this article highlight the potential adverse impact of the provision of advice from mothers on adult children’s well-being, as well as marked gender differences in the magnitude of this association, such that advice was associated with higher depressive symptoms among sons, but not daughters. Moreover, our findings suggest that emotional closeness and tension mute and exacerbate the association between advice from mothers and sons’ depressive symptoms, respectively. Taken together, these findings have important implications for theories of face, relational regulation, gender socialization, and identity by highlighting the ways in which gender and relationship quality shape the association between receiving advice and well-being. We hope that future scholars will build on this study to further our understanding of the contexts in which the provision of advice between generations can impact well-being, and how social statuses and relationship quality influence these patterns.

## Data Availability

The data used in this analysis are not publicly available. The analyses reported in this study were not preregistered.
